# Supplementary material for: Effects of word familiarity and receptive vocabulary size on speech-in-noise recognition among young adults with normal hearing
Source: PLoS One. 2022 Mar 10;17(3):e0264581. doi: 10.1371/journal.pone.0264581 (PMC8912124; doi:10.1371/journal.pone.0264581)
Supplement: S1 Table — Disyllabic target words acquired at 4 years, 9 years, 12 years, and 15 years of age. (DOCX) [file pone.0264581.s001.docx]

| **4 yrs** | **9 yrs** | **12 yrs** | **15 yrs** |
| --- | --- | --- | --- |
| angel | afghan | adder | ballast |
| apple | baboon | airmail | balsam |
| balloon | bandstand | axis | bolus |
| belly | birthstone | banknote | brioche |
| biscuit | blowfish | bigot | butane |
| candy | bronco | brisket | buzzword |
| chipmunk | buffoon | chairlift | cadre |
| circus | bullfight | chaplain | cathode |
| costume | cobbler | cherub | cloister |
| cowboy | congress | cistern | cohort |
| cupcake | courthouse | closure | couscous |
| doorknob | curveball | coffer | credo |
| dresser | dachshund | conman | cretin |
| feather | dashboard | cursor | dowry |
| freezer | detour | dockyard | duvet |
| goldfish | drainpipe | drywall | endive |
| grownup | drawbridge | facelift | escrow |
| haircut | dumpster | fauna | ethos |
| hamster | feedbag | fractal | eunuch |
| jelly | footrace | gherkin | extern |
| ketchup | forklift | groundwork | fascia |
| letter | gizmo | gumbo | fissure |
| lettuce | glowworm | hemlock | flaxseed |
| lion | greenhouse | hormone | frigate |
| mailman | headdress | icehouse | furlough |
| melon | headset | jackal | genome |
| monkey | housecoat | kingpin | grassroots |
| monster | keyword | lanyard | haggis |
| oatmeal | lakeshore | limestone | hipster |
| pancake | mantle | marrow | hummus |
| paper | minnow | mastiff | junket |
| pencil | nougat | narwhal | kava |
| penny | playoff | neuron | lambda |
| pizza | project | omen | magnate |
| playground | puma | payout | mastoid |
| pocket | quartet | pita | nexus |
| popcorn | racetrack | pumice | osprey |
| preschool | region | resin | peptide |
| rainbow | rematch | roadster | pigmy |
| rattle | reprint | schnitzel | playlist |
| sailboat | roommate | shrapnel | quorum |
| sandbox | saloon | skycap | radon |
| shoelace | seedpod | snafu | saffron |
| shoulder | shellfish | songstress | sclera |
| shower | shelter | sorbet | septum |
| sidewalk | snorkel | spectrum | stalwart |
| soda | spokesman | standpoint | stigma |
| sofa | stagecoach | steward | stopgap |
| spaceship | stardust | sunlamp | suture |
| stairway | starship | tangent | synapse |
| sticker | tailbone | trowel | syntax |
| stranger | tanker | tundra | thrombus |
| sunshine | tigress | turncoat | tincture |
| table | toadstool | vantage | tungsten |
| teacher | tollbooth | venue | umlaut |
| tiger | townsfolk | warlord | vita |
| toothbrush | trainload | wavelength | wainwright |
| turtle | trapdoor | webpage | wormwood |
| window | widow | workup | xenon |
| winter | workday | zircon | zygote |
